# Can the SASSY survey guide climate-animal health communication in veterinary clinics?

**DOI:** 10.3389/fvets.2026.1844415

**Published:** 2026-06-08

**Authors:** Rhea Amatya, Kelly Greenhut, Catherine Taylor Krouse, Caroline Kern-Allely, Danielle Scott, Kim Hillyer, Colleen Duncan

**Affiliations:** 1 College of Veterinary Medicine and Biomedical Sciences, Colorado State University, Fort Collins, CO, United States; 2 Department of Clinical Sciences, College of Veterinary Medicine and Biomedical Sciences, Colorado State University, Fort Collins, CO, United States; 3 Marketing and Communications, College of Veterinary Medicine and Biomedical Sciences, Colorado State University, Fort Collins, CO, United States; 4 Department of Microbiology, Immunology, and Pathology, College of Veterinary Medicine and Biomedical Sciences, Colorado State University, Fort Collins, CO, United States

**Keywords:** animal health, climate change, communication, environmental hazards, SASSY

## Abstract

Effective client communication requires understanding audience perspectives and values. Although many U.S. veterinary clients express concern about environmental issues, climate beliefs vary across regions and practice settings, limiting the applicability of national survey findings to individual clinics. The Six Americas Super Short Survey (SASSY) is a validated four question audience segmentation instrument that categorizes individuals into six climate belief groups. We used SASSY at our veterinary hospital to inform educational resource development. The tool proved feasible and informative, and we encourage veterinary practices to consider SASSY as a practical method for understanding client climate perspectives and guiding clinic-specific communication strategies.

## 1 Introduction

Effective climate–health messaging depends on clear communication from trusted sources. Messages tailored to specific audiences are more likely to build understanding, reduce adverse health impacts, and motivate protective behaviors. Surveys of US veterinary clients suggest that the majority are concerned about the effects of climate change on their pets and desire guidance from veterinary professionals regarding climate-related hazards, such as disasters (1, 2). However, national-level findings mask substantial variability in climate beliefs across regions, communities, and practice settings, limiting their usefulness for individual clinics. As climate-associated hazards, such as heat, air pollution, and disasters, increasingly affect animal health and welfare (3), veterinary practices need a practical approach to understand client climate perspectives to tailor communication effectively.

Audience segmentation is a communication strategy that categorizes a population into groups based on shared beliefs, values and levels of concern to guide message framing and delivery (4, 5). In the context of climate change, segmentation allows scientific information to be presented in ways that resonate with distinct belief profiles, supporting more effective engagement while preserving trust. Without insight into local audience composition, communications efforts risk being overly generalized or misaligned with client value systems. This, in turn, will make a client less likely to trust in the information and thus less likely to take the desired or necessary actions. Although widely applied in human health and environmental communication, audience segmentation has been underutilized in veterinary medicine (4, 6).

The Global Warming’s Six Americas survey was developed by the Yale Program on Climate Change Communication and the George Mason Center for Climate Change Communication to categorize the US public into distinct audience segments based on their beliefs, attitudes, and engagement with global warming in order to inform more effective climate communication strategies (7). The tool categorizes individuals into six groups, Alarmed, Concerned, Cautious, Disengaged, Doubtful, or Dismissive. Alarmed indicates the highest belief in global warming and support of climate-related political actions to address it, while Dismissive indicates the lowest belief and opposition to climate-related policies. A four-question subset of the full instrument, the Six Americas Super Short survey (SASSY), classifies respondents with approximately 70% accuracy relative to the complete survey (8, 9). This tool has been used to inform climate communication across diverse sectors (10–12). To our knowledge, SASSY has not been applied in veterinary clinical settings. Here, we describe our experience piloting SASSY in a veterinary hospital and consider its potential utility for guiding climate-animal health communication in veterinary medicine more broadly.

## 2 SASSY survey pilot project

We conducted an anonymous survey of veterinary clients at Colorado State University (CSU)’s Veterinary Health System (VHS) from October 16th, 2025, to January 16th, 2026. The survey (Supplementary material 1) included the four SASSY questions (How important is the issue of global warming to you personally? How worried are you about global warming? How much do you think global warming will harm you personally? How much do you think global warming will harm future generations of people?), one on the species of animal they had brought to the clinic, one on which environmental hazards they worry about for their pet, and two on their preferred methods for messaging on climate animal health communication. Clients accessed the survey by scanning a QR code displayed on notices in waiting and exam rooms throughout the small animal, equine, and livestock buildings, which directed them to the survey using Qualtrics (Qualtrics, Provo, UT). The respondents needed to be at least 18 years of age, a current client of CSU’s VHS, and provide consent to participate. Responses to the four SASSY questions were analyzed using the standardized SASSY Group Scoring Tool CSV template on the website (8), which segmented respondents into the 6 categories (Alarmed, Concerned, Cautious, Disengaged, Doubtful, or Dismissive). The survey was classified as exempt by the CSU Institutional Review Board.

**Figure 1:**
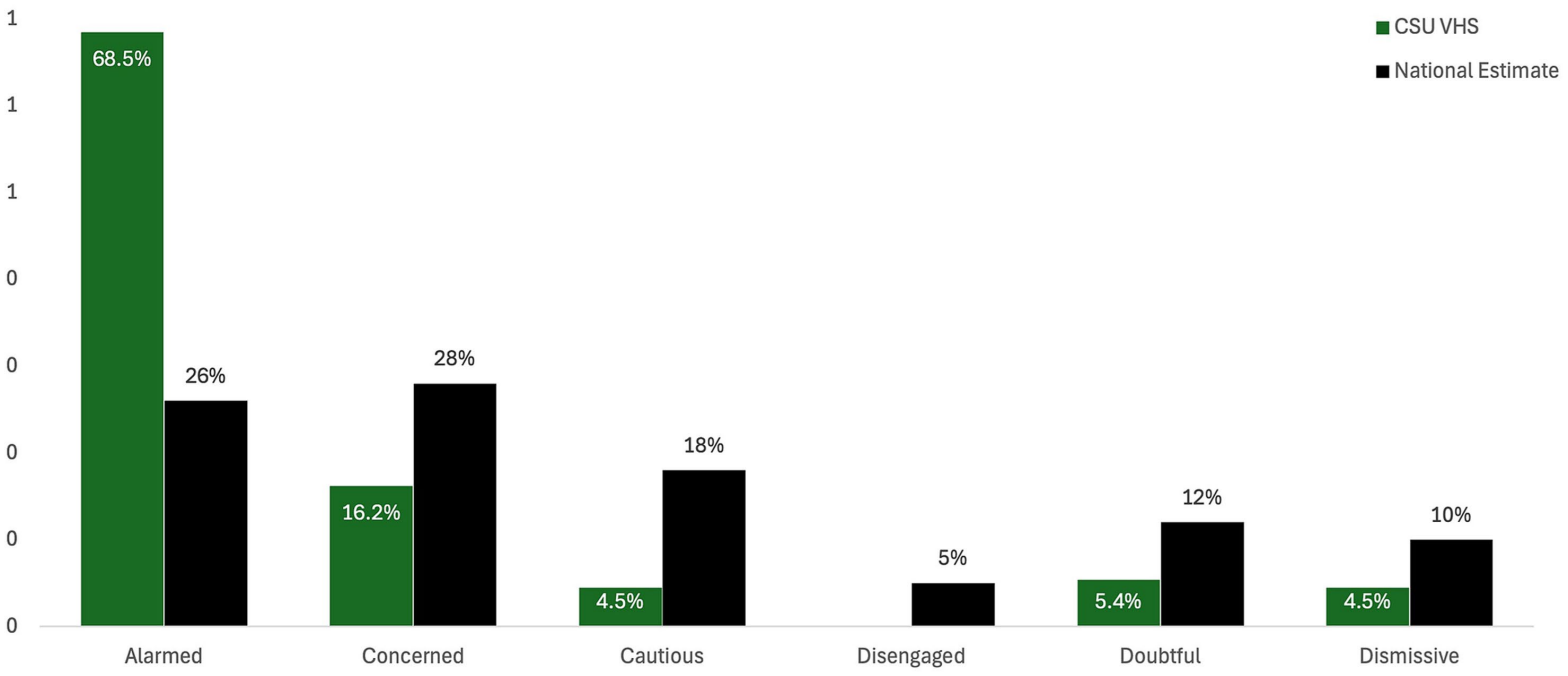
Percentage of VHS client survey respondents in the SASSY audience segmentation labels of CSU VHS clients compared to the US December 2024 national estimate.

**Figure 2:**
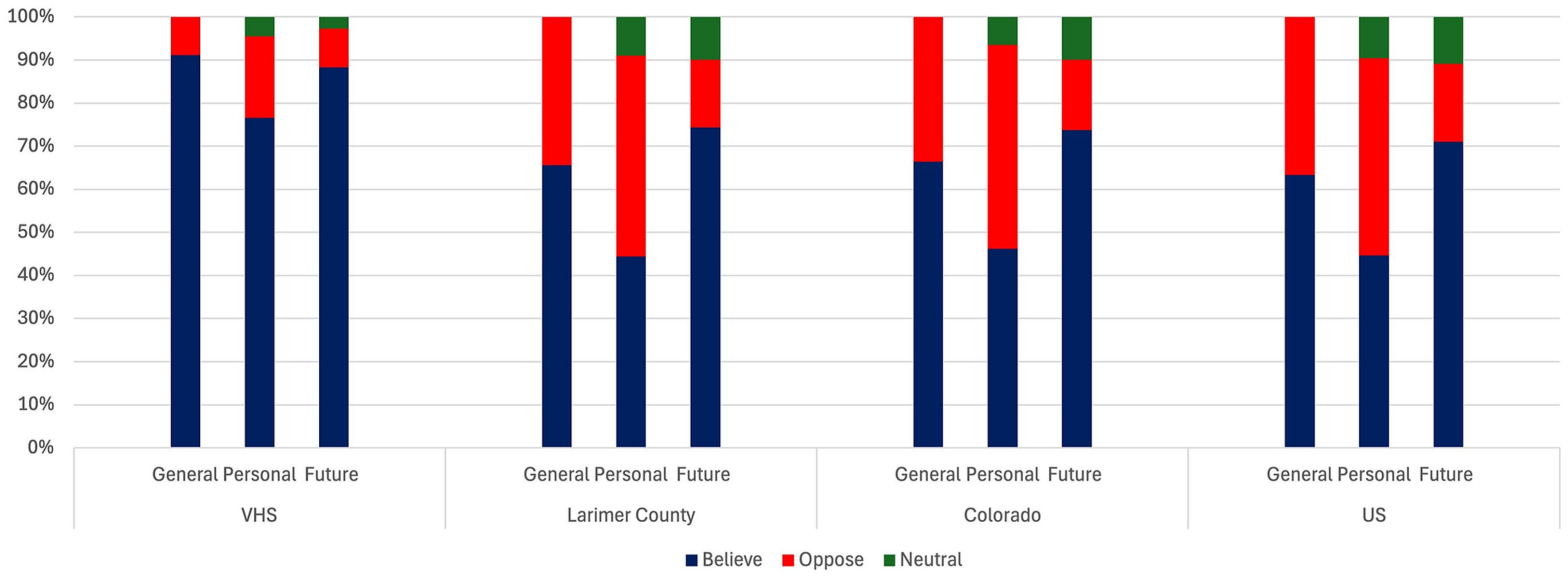
Percentage of VHS clients reporting concern about global warming: overall worry (“General”), perceived personal harm (“Personal”), and anticipated harm to future generations (“Future”) due to global warming. Responses were grouped as: (1) Believe – higher concern (e.g., very/somewhat worried, a great/moderate amount of harm), (2) Oppose – lower concern (e.g., not very/not at all worried; only a little/not at all harm), and (3) Neutral (e.g., do not know). Results are compared to Larimer County, Colorado, and U.S. reports (13, 14).

Of the 111 respondents, most were classified as Alarmed (68.5%, *n* = 76) followed by Concerned (16.2%, *n* = 17) with far fewer classified as Cautious (4.5%, *n* = 5), Disengaged (0%, *n* = 0), Doubtful (5.4%, *n* = 6) or Dismissive (4.5%, *n* = 5). Our VHS respondents were significantly more Alarmed when compared to the national average of 26% (exact binomial test, *p* < 0.001) (Figure 1). Our VHS respondents are more worried about global warming, believe that global warming will harm them personally and that global warming will harm future generations when compared to previous surveys of people in Larimer County, Colorado, and the US (Figure 2) (13, 14). Client-reported environmental concerns and preferred sources and channels for climate-related pet health communication are summarized in Supplementary material 1.

## 3 Discussion

We propose that the SASSY tool is a helpful resource to inform climate communication strategies for veterinary clients. Audience segmentation is particularly relevant in veterinary medicine because veterinary care relies on trust and informed decision-making. Unlike human health, where individuals make decisions for themselves, veterinary professionals must communicate risk to caregivers who act on behalf of animals. When discussing emerging threats such as climate-related hazards, understanding whether clients are Alarmed, Concerned, Uncertain, or Dismissive directly informs both message framing and depth of information provided, helping clinics to allocate limited educational time and resources more efficiently while maintaining alignment with client values.

At our hospital, SASSY results indicated that clients expressed greater concern about climate change relative to national and state-level surveys (13, 14), suggesting a higher readiness to engage with climate-related pet health information. These findings directly inform our institutional response. Educational materials are being developed to address client-identified priority concerns and to be distributed through client preferred communication channels. These are intended to be practical and action-oriented, including both adaptation and mitigation strategies. Our results also highlight that we should communicate about our institutional sustainability initiatives, such as messaging around CSU’s new Veterinary Hospital and Education Complex, emphasizing its pursuit of LEED and WELL certifications along with college efforts to expand sustainability education to veterinary students (15, 16).

In contrast to our primarily Alarmed clients, clinics serving populations with higher proportions of Doubtful or Dismissive clients may adopt a different framing, while maintaining identical clinical standards. In those settings, communication may lead with observable clinical trends such as increased heat-related emergencies, prolonged vector seasons, or wildfire smoke exposure rather than climate change framing, emphasizing the relevance of environmental hazards to everyday pet health. Conversely, when patient specific risk factors are present, communication can begin with those vulnerabilities. For example, brachycephalic breeds are more susceptible to heat related illness (17) and equine athletes may experience performance impacts associated with air pollution (18). In these cases, anchoring the discussion in individual patient risk provides a clinically grounded entry point for broader environmental health considerations. The medical recommendations do not change, only the framing and sequencing of information do. By grounding communication strategies in locally derived data rather than assumptions, veterinary clinics can integrate environmental health into practice in a manner that preserves trust and improves uptake of preventive guidance.

Implementing audience segmentation surveys in clinical settings requires attention to potential sources of bias and practical challenges. Survey delivery method and timing can influence who responds, and practices should aim for approaches that reach a representative cross-section of their client population rather than only those with extra time or higher baseline engagement with environmental topics. Response rates may also vary by clinic type, season, and competing demands on staff and clients. In this pilot, voluntary participation via QR code likely introduced self-selection bias, with more environmentally engaged clients disproportionately represented, and equine and livestock clients were underrepresented. These factors reinforce that our findings should be interpreted as illustrative of the tool’s application rather than as definitive characterizations of our client population. As our data collection coincided with the holiday season and a period of major construction at the hospital, we will continue to seek client feedback.

Veterinarians are consistently viewed as credible and trusted sources of animal health information and therefore have the unique opportunity to serve a key role in science communication regarding environmental health risks across species (2, 19–21). As environmental hazards increasingly influence animal health, aligning communication with client perspectives strengthens the veterinary profession’s capacity to respond to emerging risks while preserving trust and supporting informed decision making.

## Data Availability

The original contributions presented in the study are included in the article/Supplementary material, further inquiries can be directed to the corresponding author/s.
