# Supplementary material for: Can the SASSY survey guide climate-animal health communication in veterinary clinics?
Source: Front Vet Sci. 2026 Jun 8;13:1844415. doi: 10.3389/fvets.2026.1844415 (PMC13322071; doi:10.3389/fvets.2026.1844415)
Supplement: Supplementary file 1 [file Table_1.DOCX]

Supplementary Material 1: Survey Questions

**Section 1: SASSY questions**

- How important is the issue of global warming to you personally? (Response options: Extremely important - Very important - Somewhat important - Not too important - Not at all important)
- How worried are you about global warming? (Response options: Very worried– Somewhat worried - Not very worried - Not at all worried)
- How much do you think global warming will harm you personally? (Response options: A great deal - A moderate amount - Only a little - Not at all - Don’t know)
- How much do you think global warming will harm future generations of people? (Response options: A great deal - A moderate amount - Only a little - Not at all - Don’t know)

**Section 2: CSU specific client communication questions**

- What animal are you here for? (Response options: Small companion animal (dog, cat, exotic, etc) - Livestock (cow, goat, sheep, pig, etc) - Equine - Other (please specify))
- What environmental hazards do you worry about for your pet? (Select all that apply response options: Air pollution, e.g. Smoke - Extreme heat - Natural disaster, e.g. wildfires - Water borne illness, e.g. harmful algal blooms - Vector borne disease, e.g. heartworm - Other (please specify) - I don’t worry about environmental hazards)
- How would you prefer to get information about climate change as it relates to your pet’s health? (Select all that apply response options: Traditional media: newspapers (print/online), local/cable news networks - Pamphlets/brochures/handouts - Posters/flyers hung in clinics - Social media - Podcasts - Website/blogs - Webinar - Scientific journals - Family/friends - Industry group websites - Other (please specify) - I don’t seek information on this topic)
- Which of the following communications channels do you primarily use to consume climate change-related news or information? (Select all that apply response options: Traditional media: newspapers (print/online), local/cable news networks - Social media – Podcasts - Website/Blogs - Webinar - Scientific journals - Family/friends - Industry group websites - Other (please specify) - I don’t seek information on this topic)
