# Supplementary material for: Can the SASSY survey guide climate-animal health communication in veterinary clinics?
Source: Front Vet Sci. 2026 Jun 8;13:1844415. doi: 10.3389/fvets.2026.1844415 (PMC13322071; doi:10.3389/fvets.2026.1844415)
Supplement: Supplementary file 2 [file Table_2.DOCX]

Supplementary Material 2: Table

Number and percent of survey respondents indicating concern about specific environmental hazards for their pet, their preferred channels for consuming climate change related information, and their primary channels for consuming climate change related information. All questions were select-all-that-apply.

| **Question** | **Response Option** | **Count (%)** |
| --- | --- | --- |
| What environmental hazards do you worry about for your pet? | Vector borne disease | 69 (62%) |
|  | Natural disaster | 64 (58%) |
|  | Waterborne illness | 63 (57%) |
|  | Extreme heat | 62 (56%) |
|  | Air pollution | 56 (50%) |
|  | Other | 13 (12%) |
|  | Have not considered before | 6 (5%) |
| How would you prefer to get information about climate change as it relates to your pet’s health? | Scientific journals | 59 (53%) |
|  | Social media | 52 (46%) |
|  | Traditional media | 45 (40%) |
|  | Website/blogs | 44 (39%) |
|  | Posters/flyers hung in clinics | 38 (34%) |
|  | Podcasts | 34 (30%) |
|  | Pamphlets/brochures/handouts | 31 (28%) |
|  | Webinar | 23 (21%) |
|  | Family/friends | 20 (18%) |
|  | Industry group websites | 17 (15%) |
|  | Other (please specify) | 10 (9%) |
|  | I don't seek information on this topic | 11 (10%) |
| Which of the following communications channels do you primarily use to consume climate change-related news or information? | Traditional media | 68 (61%) |
|  | Social media | 61 (54%) |
|  | Scientific journals | 46 (41%) |
|  | Website/blogs | 44 (39%) |
|  | Other | 4 (35%) |
|  | Podcasts | 39 (24%) |
|  | Family/friends | 27 (14%) |
|  | Webinar | 16 (13%) |
|  | Industry group websites | 14 (12%) |
|  | I don't seek information on this topic | 13 (4%) |
